# Osmotic Dehydration with Alternative Humectants for the Production of Ultrachilled Cherry Tomatoes

**DOI:** 10.3390/foods15142569

**Published:** 2026-07-22

**Authors:** Efimia Dermesonlouoglou, Despoina Manoleri, Maria Giannakourou, Petros Taoukis

**Affiliations:** Laboratory of Food Chemistry and Technology, School of Chemical Engineering, National Technical University of Athens, Iroon Polytechneiou 9, Zografou, 15780 Athens, Greece; efider@chemeng.ntua.gr (E.D.); despmanoleri@gmail.com (D.M.); mgian@chemeng.ntua.gr (M.G.)

**Keywords:** *S. lycopersicum* var. *cerasiforme*, osmotic dehydration, humectants, water activity, freezing point, mass transfer, ultrachilled storage

## Abstract

The aim of the study was to explore and develop an innovative food category with distinct physicochemical properties (reduced water activity, a_w_ ≤ 0.9) and a lower initial freezing point (IFP), in the range −10 °C to −8 °C, by integrating mild dehydration techniques (osmotic dehydration (OD)), with the scope of ensuring superior quality until final consumption. In this context, peeled cherry tomatoes were OD-treated at 35 °C for up to 180 min in hypertonic solutions, consisting of alternative humectants (maltitol, oligofructose, glycerol, glycine, alanine, and their combinations, as well as NaCl and CaCl_2_). Mass transfer (water loss (WL), solid gain (SG)), water activity (a_w_), IFP, and sensory properties’ changes were monitored during the osmotic process. The most effective osmotic solutions, regarding a_w_ decrease, as well as IFP depression, were glycerol (50%), glycine (11%)–glycerol (39%) and alanine (7%)–glycerol (43%). a_w_ and IFP of OD tomatoes (120 min −35 °C) significantly decreased (up to 0.9063 compared to 0.9778, and up to −7.55 °C) compared to −1.05° C of the untreated sample.

## 1. Introduction

Osmotic dehydration (OD) consists of immersing foods in hypertonic sugar and/or salt solutions to partially remove water without phase change through water diffusion from the tissue, solute uptake into the matrix, and limited leaching of native components [1,2]. The main objective is to reduce water activity (a_w_), while the efficiency of the process depends on tissue characteristics and processing variables, including solute concentration and molecular weight, temperature, immersion time, and agitation [2,3]. OD is particularly suitable for thermosensitive plant tissues, and it also enables selective solute incorporation, supporting targeted modification of nutritional, textural, and sensory properties [4,5,6,7]. However, the achieved reduction in water activity is not sufficient for microbiological stability, leading to the need for complementary preservation techniques (e.g., chilling or freezing). When combined with freezing, OD has led to the development of frozen foods of a_w_ 0.8–0.9, also known as osmodehydrofrozen (ODF) products [8]. Osmotic pretreatment depresses the initial freezing point while increasing the glass transition temperature, thereby improving structural stability during frozen storage. Consequently, ODF enhances quality retention, reduces the amount of freezable water, and lowers energy consumption, refrigeration load, and storage costs [9].

To balance functionality and sensory quality, combinations of humectants or alternative agents are often used. Having low molecular weights and high solubilities, common humectants, such as glycerol, sugars, and salts, lower water activity, act as cryoprotectants and improve texture, though high levels may negatively affect the sensory properties (aroma/flavor, taste). More specifically, glycerol, a sugar alcohol with low sweetness, has been used as the main a_w_-lowering agent during OD [10,11]. Amino acids and protein hydrolysates have shown strong a_w_-lowering effects, with L-arginine, L-lysine, L-ornithine, L-proline, glycine, or mixtures of amino acids being particularly effective and generally not negatively affecting flavor (with possible exceptions for arginine and certain hydrolysates). Other novel humectants, including low-molecular-weight carbohydrates such as trehalose, oligofructose, and oligogalactose (e.g., tagatose), have been employed due to their “generally accepted as safe” GRAS status and functional or prebiotic benefits [12,13]. Beyond carbohydrates, sugar alcohols, and amino acids, increasing attention has focused on low-cost agro-industrial by-products as sustainable osmotic agents, supporting circular-economy and cost-reduction strategies. Sugar beet molasses has been successfully applied to the osmotic dehydration of apples and celery root, while prickly pear molasses has been proposed as a sucrose substitute for orange dehydration, improving dehydration efficiency and antioxidant retention [14,15,16,17]. Dairy by-products have also shown promising results: hydrolyzed cheese whey permeate has been used for peppers, whereas whey combined with sucrose and salt increased tomato dehydration efficiency by up to 65% while generating several value-added products [18,19]. Other unconventional osmotic media, including jaggery, cane molasses, honey, and concentrated fruit juices or syrups, have also demonstrated satisfactory osmotic performance with additional nutritional and sensory benefits. Overall, these studies highlight agro-industrial by-products as promising sustainable alternatives to conventional humectants, although their variable composition requires optimization of processing conditions and careful evaluation of their effects on product quality.

Among alternative subzero preservation techniques, superchilling involves partial water crystallization (approximately 5–30%) while maintaining the product temperature 1–2 °C below its initial freezing point [20]. The formation of a thin surface ice layer acts as a thermal buffer and may enhance mechanical strength; however, progressive ice growth during storage can induce cellular damage [20,21]. Although widely studied in fish and poultry, applications in plant products remain limited, due to their sensitivity to chilling and freezing injury [21]. Compared with refrigeration, the benefit of superchilling is shelf-life extension, whereas energy requirements are significantly lower, when compared to frozen food counterparts [22]. Current energy-saving strategies, including moderate increases in frozen storage requirements (e.g., from the current legislation requirement of −18 to the proposed −15 °C) further highlight the industrial relevance of subfreezing technologies, despite the risk of large ice crystal formation [23]. Supercooling, on the other hand, maintains foods in an unfrozen state below their initial freezing point (typically −1 to −3 °C), inhibiting microbial and oxidative deterioration without ice-induced structural damage [24,25,26]. Nevertheless, its practical implementation is constrained by the narrow stability range of the metastable state, which is strongly influenced by composition, moisture distribution, protein characteristics, and microbial load [17,18,19,20,21,22,23,24,25,26,27,28,29]. Ultrachilling or ultrachilled storage follows a similar concept, combining rapid cooling close to the freezing point with slow temperature reduction to preserve freshness under non-frozen conditions, avoiding the detrimental effects of ice crystallization on food quality [30]. This method can be used in conjunction with ordinary cryogenic distribution and transportation methods to supply consumers with high-quality fresh-like foods.

Cherry tomatoes are nutrient-rich and bioactive, valued for their small size, flavor, and diverse colors, and are consumed both fresh and in processed products [31]. Their thin skin and high juiciness make them highly perishable, requiring effective processing or packaging to extend shelf life [32,33]. Conventional freezing alone provides limited quality retention, particularly for direct consumption. However, dehydrofreezing—partial dehydration prior to freezing—has been shown to be an effective alternative for preserving cherry tomatoes [6,11,34,35].

Despite the extensive literature on osmotic dehydration as a pre-treatment for freezing (dehydrofreezing), most studies optimize humectant selection solely with respect to water activity reduction, treating the effect on the initial freezing point (IFP) as a secondary or unreported outcome. Consequently, there is limited systematic knowledge on how different humectants and their combinations simultaneously affect a_w_ and IFP, which is the key coupled property needed to design a genuinely non-frozen, ultrachilled product rather than a conventional dehydrofrozen one. The aim of this study was to produce partially dehydrated cherry tomatoes with reduced water activity (a_w_) and a depressed initial freezing point (IFP), through osmotic dehydration with alternative humectants, so as to enable a dual-step preservation strategy–osmotic processing followed by ultrachilled storage at temperatures between conventional refrigeration and freezing. To achieve this, glycerol, glycine, alanine, maltitol, oligofructose, and their combinations were used as osmotic solutes. Specifically, mass exchange, a_w_, IFP, color, and total sensory quality were evaluated during OD at 35 °C for 30–180 min, and the effect of two selected OD treatments on the shelf-life stability of cherry tomatoes during ultrachilled storage was subsequently investigated, thereby moving beyond mass-transfer characterization toward process-to-storage design.

## 2. Materials and Methods

### 2.1. Sample Preparation

Cherry tomatoes (*S. lycopersicum* var. *cerasiforme*) (diameter: 30 ± 10 mm) were purchased at the local market and kept in temperature-controlled cabinets for a maximum of 1 day (approximately 4.0 °C). Before osmotic treatment, tomatoes were washed with tap water and then blanched in water at 80 °C for 30 s, for both peel removal and enzyme inactivation [11]. For the raw material (cherry tomatoes), several physicochemical parameters were measured, namely a_w_, total soluble solids (Brix), the initial freezing point (IFP), and color parameters.

### 2.2. Osmotic Dehydration

Osmotic dehydration (OD) was carried out in solutions by dissolving the humectant with the following concentrations (C): maltitol 30–50% *w*/*w* (MALTIDEX CH 16385, Cargill, Minneapolis, MN, USA), oligofructose (Fructooligosaccharides, GOFOS P95, Norkem Limited, Cheshire, UK) 40–60% *w*/*w*, glycerol (Glycerin 99.5%, Falcon S.A., Athens, Greece) 40–60% *w*/*w*, glycine 18.7% *w*/*w* (Glycine 99%, Thermo Fisher Scientific Inc., Waltham, MA, USA), alanine 13.7% *w*/*w* (L-Alanine 99%, Thermo Fisher Scientific Inc., Waltham, MA, USA), or their combinations, glycine–glycerol 11–39% *w*/*w*), alanine–glycerol 7–43% *w*/*w*, and trehalose–oligofructose 17–33% *w*/*w*, 3.5% NaCl and 1.5% CaCl_2_ ($CaCl_{2}$ dihydrate, CASO Food Flakes, Solvay Chemicals, Tuscany, Italy), at (T) 35, 45, 55 °C for time duration (t) up to 180 min to yield a solid to liquid ratio of 1:5 (*w*/*w*) [11,36] (Table 1).

Sodium chloride was included in the osmotic solution to balance the slight sweetness obtained during osmotic pretreatment, as well as to increase the driving force of the process [37]. Calcium chloride was used to retain or even to improve the texture properties of the osmodehydrated product [38]. Tomatoes were pre-weighed, put in cylindrical glass containers and placed in water baths of controlled temperature (Grant GL5400 Linear Shaking Water Bath, Royston, UK) under constant agitation (240 rpm). At times (t) 15, 30, 60, 90, 120, 150 and 180 min, samples (triplicates) were removed from jars and weighed after carefully blotting the excess coating solution. To deal with potential sample buoyancy, a mesh was placed over the samples inside the glass container to keep them submerged during all the time of the osmotic procedure.

#### 2.2.1. Water, Total Solids, Total Soluble Solids and Water Activity

Water content and total solids (TS) were monitored during the process, measured by drying at 105 °C for 24 h according to the AOAC method (1990). Total soluble solids (TSS) were measured on homogenized samples using a benchtop Abbe refractometer and were expressed as °Brix. Water activity (a_w_) was monitored using Aqua LAB 4TEV (Decagon Devices, Inc., Pullman, WA, USA). Prior to measurement all samples were equilibrated at room temperature. Readings were obtained after adequate equilibration of the samples in the device after 15–20 min.

#### 2.2.2. Determination of Initial Freezing Temperature

Temperature thermocouples (PC208W Datalogger, Campbell Scientific, Inc., Leicestershire, UK, precision: ±0.1 °C) were inserted into the thermal center of each tomato sample (five samples for each condition). Temperature was monitored during the freezing process, and the freezing curve was constructed. The determination of the initial freezing temperature or “initial freezing point” (IFP, T_f_) was based on the water–ice transition during cooling where supercooling (SC) occurs. The IFP was derived from the relatively long temperature plateau on the cooling curve (following SC) where latent heat is released as ice forms [39].

#### 2.2.3. Mass Transfer

Water loss (WL) and total solids gain (SG), which describe the mass transfer phenomena during the OD process, were calculated (Equations (1) and (2)):

(1) $$WL=\frac{\left(M_{0}-m_{0}\right)-\left(M-m\right)}{m_{0}}$$

(2) $$SG=\frac{\left(m-m_{0}\right)}{m_{0}}$$

where M_0_ and m_0_ are the initial total mass and dry mass of the tomato sample at OD time zero, respectively, and M and m are the total mass and dry mass of the tomato sample at OD time t, respectively. Then, WL and SG can be described as a function of the square root of time (Equation (3) [36]):

(3) $$WL\;or\;SG=k_{x/SG}\sqrt{t}$$

where WL in g water/g dry solid, SG in g solids/g initial dry matter, and k_x_ is the water loss constant (k_WL_) or the total solids uptake constant (k_SG_).

#### 2.2.4. Determination of Tomato Color

Objective color was measured on the surface of the tomatoes using an Xrite-i1 portable digital colorimeter (Gretag-Macbeth, Grand Rapids, MI, USA) and expressed in the CIE-Lab scale. Color was measured at three equidistant equatorial points on the tomatoes on at least five replicates. The total color change during the OD process is mathematically described by Equation (4) [40].

(4) $$\Delta Ε=\sqrt{{\left(L-L_{o}\right)}^{2}+{\left(\alpha -\alpha_{o}\right)}^{2}+{\left(b-b_{o}\right)}^{2}}$$

where L (lightness), a (from +: red to −: green), b (from +: yellow to −: blue), and L_0_, a_0_, b_0_ values at time t (of the OD process) and at time zero (0), respectively. Tomato color was also expressed using TCI (Total Color Index) index, given by the following equation (Equation (5)) [41].

(5) $$TCI=\frac{\alpha }{b}$$

which is an indicator that describes the color change in tomato fruit.

#### 2.2.5. Sensory Evaluation of Tomato

Sensory evaluation was carried out by a panel of eight to ten trained assessors of the sensory laboratory of NTUA, selected and trained according to ISO 8586:2023 [42], following the general sensory evaluation methodology of ISO 6658:2017 [43].and the quantitative scaling guidelines of ISO 4121:2003 [44],on the following attributes: glossiness, deformation/shrinkage, red color for appearance, firmness and juiciness for texture, sweetness, sourness for flavor and aftertaste, tomato aroma. The intensity was evaluated using a 9-point scale (1 = low intensity and 9 = high intensity). The panel also performed an overall impression test, using a 9-point hedonic scale (1 = extremely dislike to 9 = extremely like) (Table 2). Regarding the ethics statement during sensory testing, the following procedure was considered. Only adults participated in the recruitment to the sensory team. Participation in the tests and assessments was voluntary. Using an appropriate form, written consent was obtained from the participants in the sensory evaluation study. Each of them could withdraw their consent without providing any justification. Each participant also consented to the processing of their personal data in accordance with Article 6 of Regulation (EU) 2016/679 of the European Parliament and of the Council of 27 April 2016 on the protection of natural persons regarding the processing of personal data and on the free movement of such data, and repealing Directive 95/46/EC (General Data Protection Regulation). All participants obtained a detailed description of the test and were informed about the food samples that would be assessed. Each of the assessors was obliged to report any indispositions and allergies and if such was the case, the subject did not participate in the tests.

#### 2.2.6. Statistical Analysis

All experiments were performed at least in duplicate, and the data were expressed as mean ± standard deviation. The significance level for analysis of variance (ANOVA) was set at *p* < 0.05 using Statista 7.0 (StatSoft, Inc., Tulsa, OK, USA). As a post hoc analysis, Duncan’s multiple range test was used to separate means with significant differences.

### 2.3. Shelf-Life Estimation of OD-Treated Ultrachilled Cherry Tomatoes

In order to comparatively estimate the shelf life of OD-treated ultrachilled cherry tomatoes vs. the untreated (control) ones, two alternative OD treatments were selected, targeting at two different T_f_ levels of −4 °C and −8 °C. Specifically, part of tomatoes were osmotically treated with glycine/glycerol-based solution at 35 °C for 72 min (OD1), and part of tomatoes were osmotically treated with the glycerol-based solution at 35 °C for 131 min (OD2). The OD solutions were selected from the previous results presented in this work, based on the sensory evaluation results, as the optimal solutions. The water activity (a_w_) of the final products ranged between 0.8957 and 0.9173. OD1 and OD2 as well as non-OD (control) samples were stored at a temperature range from 6 to −6 °C (ultrachilled storage conditions), depending on their T_f_ value (non-OD: 2 °C, 6 °C; OD1: −2 °C, 2 °C, 6 °C; OD2: −6 °C, −2 °C, 2 °C). The scope was to store the products at temperatures above their initial freezing point, T_f_. The samples were sensorially tested at regular times, as described in Section 2.2.5.

## 3. Results and Discussion

### 3.1. Physicochemical Properties of the Raw Material

The water activity (a_w_) of the raw material was measured at 0.977 ± 0.010, water content was 0.93 ± 0.01 g/g of sample, total soluble solids (TSS) were 7.5 ± 0.1 °Brix and pH was 4.515 ± 0.076 (mean ± standard deviation; five samples). Similar values were reported by Azoubel and Murr (2004), Dermesonlouoglou et al. (2024), and Tsouvaltzis et al. (2023) [11,45,46]. More specifically, cherry tomatoes were selected at this high content of total soluble solids (around 7.0 °Brix), as this level is commonly perceived as a good predictor for both ripeness and high sensory quality [31]. The color was characterized by L value of 34.36 ± 0.78, a value of 16.50 ± 2.23 and b value of 1.21 ± 0.09 (a/b: 1.21 ± 0.09) (similar to Tsouvaltzis et al., 2023) [46]. The initial freezing temperature was determined to be −1.06 ± 0.06 °C, which is within the range of −1.67 and −0.89 °C reported for tomatoes [47].

### 3.2. Assessment of OD Process

**Figure 1:**
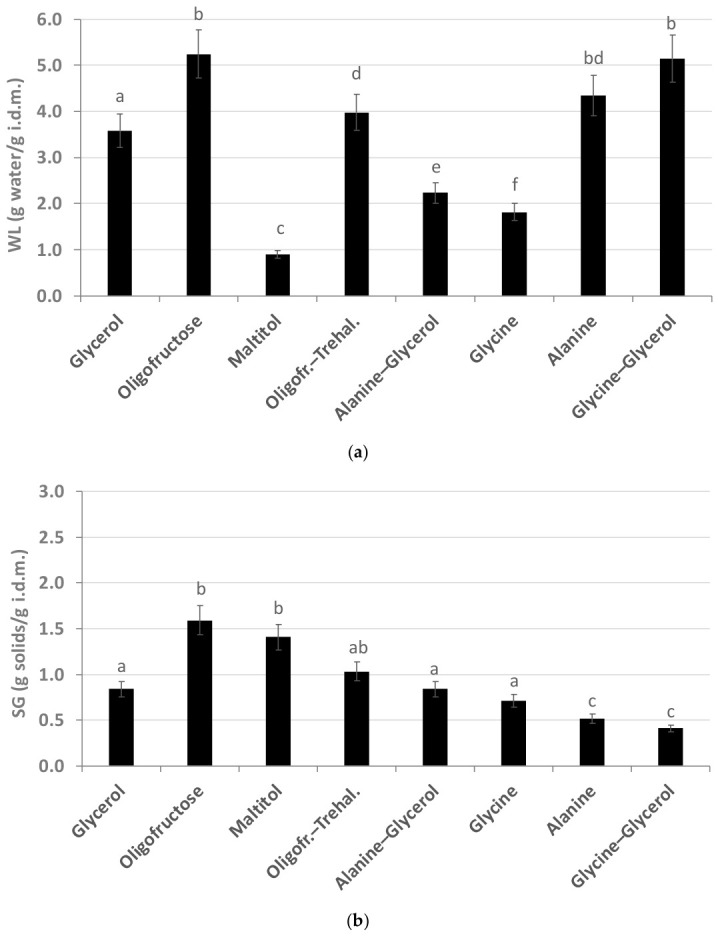
(**a**) Water loss (WL) and (**b**) solid gain (SG) of OD cherry tomatoes at 35 °C for 120 min using (*w*/*w*) 50% glycerol, 50% oligofructose, 50% maltitol, 17% trehalose/33% oligofructose, 13.6% alanine, 18.6% glycine, 7% alanine/43% glycerol, 11% glycine/39% glycerol solution. (Experimental data points: Average ± standard error. Different letters in each figure (a–f) indicate significant differences, calculated by Duncan’s multiple range test for a significance level of *p* = 0.05).

The results for the OD of cherry tomato samples (temperature 35 °C and immersion time 120 min), in terms of water loss (WL) and solid gain (SG), are presented in Figure 1a,b. The greatest water loss was obtained with oligofructose, maltitol, and the glycine–glycerol mixture (5.24 ± 0.14, 4.90 ± 0.09, and 5.13 ± 0.05 g H_2_O/g d.w., respectively). Significant losses were measured for alanine–glycerol, oligofructose–trehalose, and glycerol treatments (4.34 ± 0.26, 3.98 ± 0.36, and 3.58 ± 0.20 g H_2_O/g d.w., respectively), whereas samples treated with (sole) amino acids exhibited lower water removal (≈2 g H_2_O/g d.w.), likely reflecting the lower solute concentrations applied. This pattern did not align with the reduction in water activity, as will be described later in this section.

Similar to water loss, solids uptake varied according to the osmotic solution applied. The highest values were recorded for oligofructose and maltitol treatments (1.59 ± 0.09 and 1.41 ± 0.11 g solids/g d.w., respectively), indicating that, despite their relatively high molecular weight, they were able to penetrate plant tissue. Nevertheless, compared with lower-molecular-weight compounds, the number of molecules entering the tissue is likely reduced. Moderate solids uptake was recorded for oligofructose–trehalose, alanine–glycerol, and glycerol solutions (1.03 ± 0.07, 0.84 ± 0.02, and 0.84 ± 0.01 g solids/g d.w., respectively), whereas the lowest uptake values were observed for treatments with glycine, alanine, and the glycine–glycerol mixture (0.71 ± 0.13, 0.52 ± 0.06, and 0.41 ± 0.19 g solids/g d.w., respectively). The differences in SG among the alternative osmotic solutions investigated can be attributed to the combined effect of solute molecular weight, solution viscosity, and the resulting diffusivity, which, as a combination of factors, govern the ease of solute penetration into plant tissue. According to Rastogi et al. [48], the diffusion coefficient of an osmotic solution is inversely related to its viscosity, so that more viscous solutions present a greater mass-transfer barrier, limiting solute uptake even under a favorable concentration gradient. This is consistent with the comparatively low SG obtained here for glycine, alanine, and their mixtures with glycerol, which were applied at lower concentrations (13.6–18.7% *w*/*w*) than the sugar-based solutions (50% *w*/*w*), and is further supported by the low k_SG_ values calculated for these treatments. Conversely, oligofructose and maltitol exhibited the highest SG despite their relatively higher molecular weight, suggesting that, at the high concentrations applied, the steep initial concentration gradient can outweigh the increased diffusional resistance associated with larger solute size, in agreement with Cichowska et al. [49], who similarly reported that molecular weight alone does not reliably predict solute uptake during OD of plant tissue. Additionally, part of the apparent SG measured for the more viscous sugar-based solutions may reflect solute adhesion at the tissue surface rather than true intracellular penetration, a phenomenon previously discussed as a confounding factor in SG determination for viscous osmotic media [50]. Overall, these results support the view that SG in OD is governed less by a single solute property than by the interplay of concentration, viscosity, and the specific solute–tissue interaction [2].

Compared to water loss, solids uptake proceeded at a slower rate, as the quantities of solutes incorporated after 2 h of treatment remained comparatively limited for the osmotic solutions used. This observation is consistent with previous reports, indicating that water transfer from the product to the surrounding medium occurs more rapidly than the counter-diffusion of dissolved solids into the tissue [11,40,41]. From a technological standpoint, the production of high-quality osmotically dehydrated products generally aims to maximize water loss while minimizing solids uptake. Maximizing water loss is essential because it is the primary mechanism by which OD reduces water activity and depresses the freezing point (Section 3.1), directly determining the extent of microbial and enzymatic stabilization achieved and, in this study, the depth of the ultrachilled storage range attainable. In this context, “solids uptake” refers specifically to the counter-diffusion of the dissolved osmotic solutes themselves (sugars, sugar alcohols, amino acids and salts from the hypertonic solution) into the plant tissue, and not to oil or fat absorption, which is not relevant to this aqueous immersion process. Minimizing this solute uptake is desirable because excessive solids incorporation can alter the tissue’s native sugar-to-acid ratio and nutrient and sensory profiles, contribute unwanted calories, and promote case-hardening or textural toughening at the tissue surface, thereby compromising the sensory and nutritional integrity of the original raw material [51].

**Figure 2:**
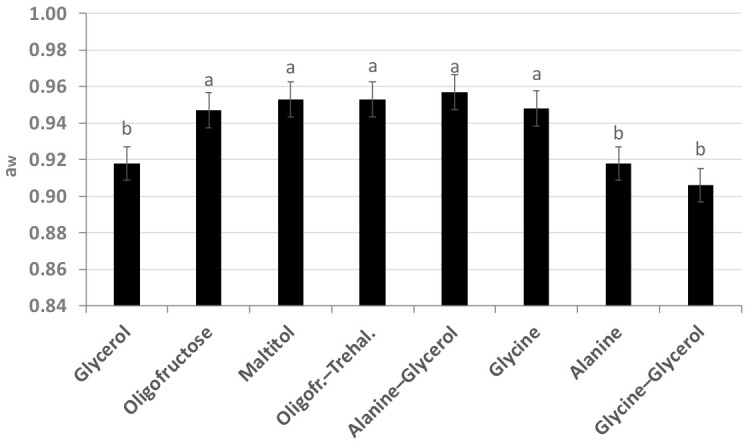
Water activity (a_w_) of OD cherry tomatoes at 35 °C for 120 min using (*w*/*w*) 50% glycerol, 50% oligofructose, 50% maltitol, 17% trehalose/33% oligofructose, 13.6% alanine, 18.6% glycine, 7% alanine/43% glycerol, 11% glycine/39% glycerol solution. (Experimental data points: Average ± standard error. Different letters (a,b) indicate significant differences, calculated by Duncan’s multiple range test for a significance level of *p* = 0.05).

Water activity values of OD cherry tomato samples (temperature 35 °C and immersion time 120 min) are presented in Figure 2. The water activity of tomato samples decreased after osmotic dehydration, with the greatest reductions observed for treatments in glycerol, glycine–glycerol, and alanine–glycerol solutions. In these cases, water activity decreased from 0.9778 ± 0.0104 to 0.9181 ± 0.0057, 0.9187 ± 0.0070, and 0.9063 ± 0.0011, respectively. Glycerol is widely used as an effective osmotic agent [11,36] because of its strong water activity-reducing capacity, cryoprotective properties, and beneficial effects on texture; accordingly, glycerol-containing solutions exhibited the highest effectiveness. Due to their low molecular weight, glycine and alanine can diffuse into plant tissues and enhance dehydration when combined with glycerol. When applied alone, however, they were less effective (a_w_ = 0.9478 ± 0.0055 and 0.9570 ± 0.0063), likely owing to the lack of synergistic interaction with glycerol and their limited solubility, which restricts attainable solution concentrations. Conversely, higher-molecular-weight solutes such as oligofructose, trehalose, and maltitol have reduced diffusivity within plant matrices, limiting osmotic efficiency [49,52]. The water activity values were calculated as follows: 0.9467 ± 0.0060, 0.9531 ± 0.0067, and 0.9532 ± 0.0059 for oligofructose, maltitol, and oligofructose–trehalose solutions, respectively, Treatments that most effectively decreased a_w_, namely glycerol and its mixtures with amino acids, did not coincide with those causing the highest water losses. These findings indicate that a_w_ decrease is governed not only by the extent of water removal, but also by the osmotic medium’s capacity to bind water and thereby reduce the fraction of free water available within the system.

**Figure 3:**
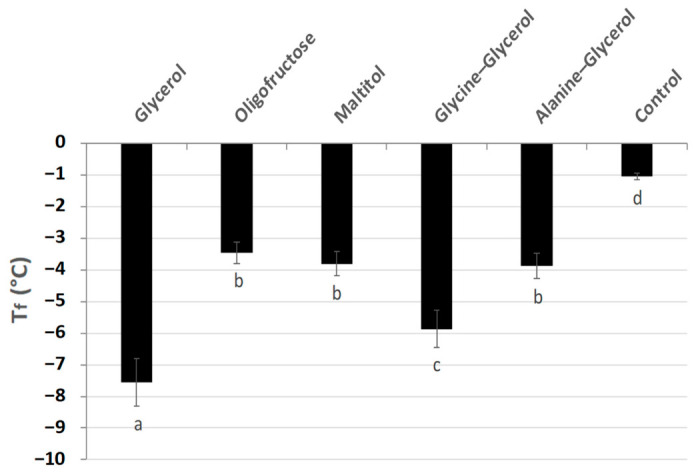
Initial freezing temperature (IFP, T_f_, °C) of OD cherry tomatoes at 35 °C for 120 min using (*w*/*w*) 50% glycerol, 50% oligofructose, 50% maltitol, 18.6% glycine, 7% alanine/43% glycerol, 11% glycine/39% glycerol solution. (Experimental data points: Average ± standard error. Different letters (a–d) indicate significant differences, calculated by Duncan’s multiple range test for a significance level of *p* = 0.05).

The results for the initial freezing point (IFP) of OD cherry tomato samples (temperature 35 °C and time 120 min) are presented in Figure 3 and Table 3 (where T_f_ values for different concentrations of the selected osmotic solutes are included). The osmotic dehydration at 35 °C for 120 min significantly reduced the initial freezing temperature for all OD-treated samples (T_f_ control = −1.06 ± 0.06 °C). The greatest freezing temperature depression was calculated for the glycerol-treated samples (−7.55 ± 1.64 °C), followed by the glycine–glycerol-treated samples (−5.87 °C), whereas oligofructose-, maltitol-, and alanine–glycerol-treated samples led to a reduction to ≈−3.7 °C. The differences in IFP depression among treatments can be rationalized by the colligative nature of freezing-point depression, which is governed by the molal concentration of solute dissolved in the tissue’s aqueous phase rather than by the absolute mass of solute taken up (SG) [53,54]. Since depression per mole of solute is independent of its chemical identity, a solute of low molecular weight will produce a substantially greater molal (and hence cryoscopic) effect than an equal mass of a higher-molecular-weight solute—a relationship formalized by Chen through the concept of “effective molecular weight” derived from freezing-point depression data of foods and aqueous solutions [54]. This explains why glycerol (MW 92 g/mol), despite a moderate SG (0.84 g solids/g d.w., Figure 1b), produced the largest IFP depression (−7.55 °C), and why the glycine–glycerol mixture achieved the second-largest depression (−5.87 °C) despite exhibiting the lowest SG of all treatments (0.41 g solids/g d.w.)—the low molecular weight of both glycine (75 g/mol) and glycerol maximizes the molal concentration achieved per unit mass of solute incorporated. Conversely, maltitol (344 g/mol) and oligofructose (average MW ~500–1000 g/mol, depending on degree of polymerization) produced the smallest IFP depressions (≈−3.7 to −4.1 °C) despite the highest SG values recorded (1.41 and 1.59 g solids/g d.w., respectively, Figure 1b); their larger molecular size dilutes the molal effect per gram of solute taken up, consistent with reports that low-molecular-weight carbohydrates and polyols are markedly more effective at depressing freezing point, gram for gram, than larger saccharides.

The magnitude of freezing-point depression calculated in the present work also compares favorably with values reported for osmodehydrofrozen fruit using conventional sucrose-based solutions. For example, Ayala-Aponte and Sánchez-Tamayo [55] reported an IFP depression from about −1.5 °C (fresh) to only −1.71 to −2.43 °C in papaya osmodehydrated with 45–55 °Brix sucrose, broadly in line with the modest depressions typical of natural fruit freezing points (apple, pear and grape range approximately −2.0 to −3.4 °C, [56]. This further supports that solute molecular weight, not solution concentration alone, is the primary determinant of achievable IFP depression, and provides additional justification for selecting low-molecular-weight humectants (glycerol, amino acids) in the present study to reach the more pronounced depressions (up to −7.55 °C) required for the ultrachilled storage strategy targeted here.

**Figure 4:**
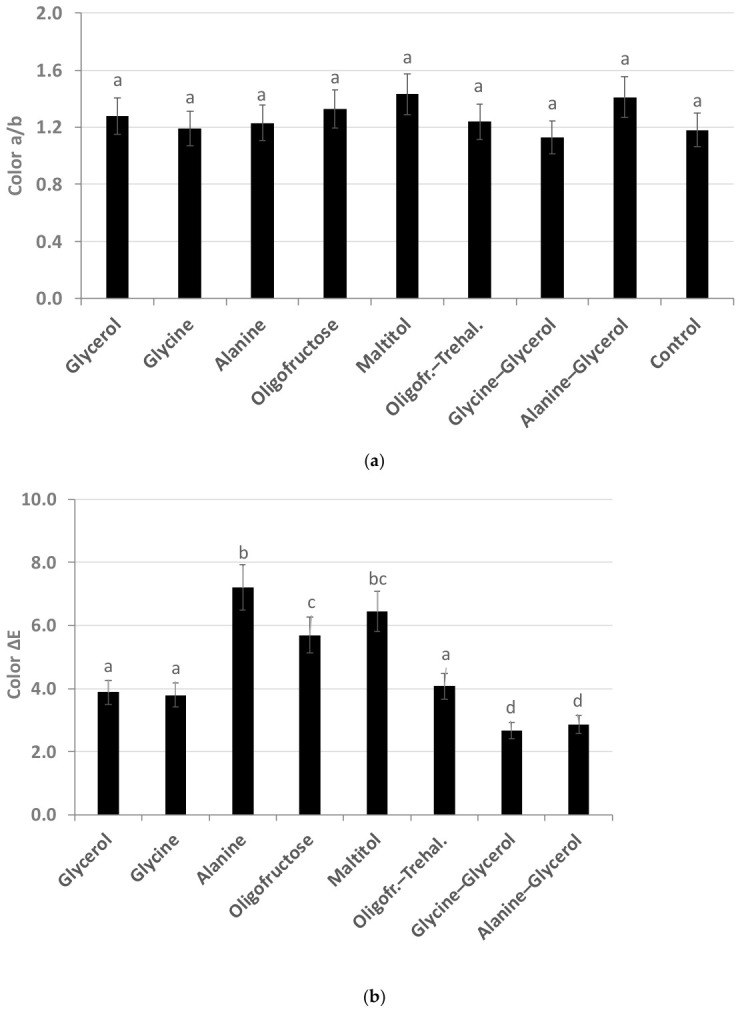
(**a**) TCI index (a/b) and (**b**) (ΔΕ) of OD cherry tomatoes at 35 °C for 120 min using (*w*/*w*) 50% glycerol, 50% oligofructose, 50% maltitol, 17% trehalose/33% oligofructose, 13.6% alanine, 18.6% glycine, 7% alanine/43% glycerol, 11% glycine/39% glycerol solution. (Experimental data points: Average ± standard error. Different letters (a–d) indicate significant differences, calculated by Duncan’s multiple range test for a significance level of *p* = 0.05).

The results for the color, expressed as a/b and ΔΕ, of OD cherry tomato samples (temperature 35 °C and immersion time 120 min) are presented in Figure 4a,b. Based on the results, the TCI index increased after the osmotic dehydration in the solutions used, apart from the alanine–glycerol and oligofructose solutions. The initial index value was 1.18 ± 0.09, whereas the maximum recorded value reached 1.41 ± 0.05. This increase can be attributed to product concentration resulting from water removal, which increased the relative lycopene content and consequently enhanced the measured red coloration [31].

**Figure 5:**
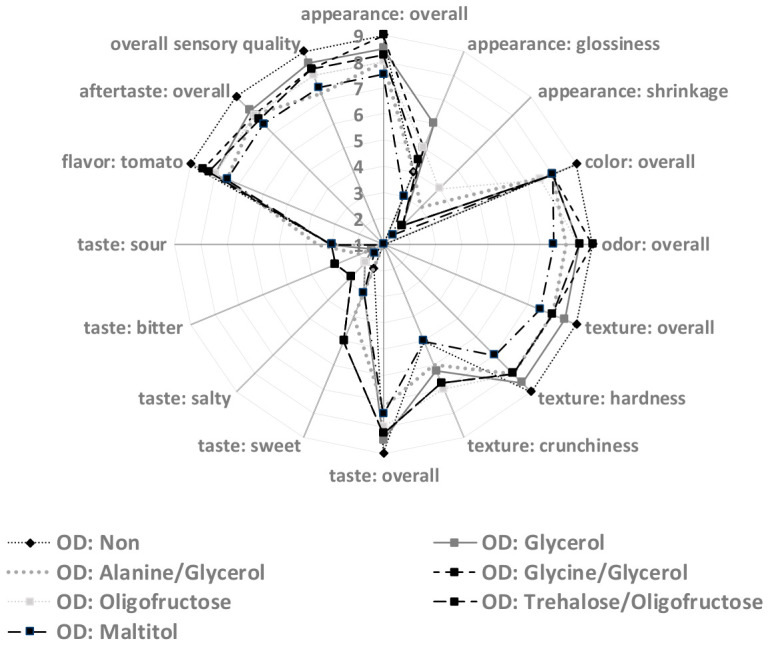
Sensory evaluation test scores: Intensity (1–9) scores for appearance (glossiness, shrinkage), texture (hardness, crunchiness), taste (sweet, salty, bitter, sour, tomato), and overall sensory quality (hedonic scale 1–9) scores for appearance, color, odor, texture, taste and overall sensory quality of tomatoes. Samples: Non-OD, OD/glycerol, OD/alanine/glycerol, OD/glycine/glycerol, OD/oligofructose, OD/trehalose/oligofructose, OD/maltitol.

Sensory evaluation showed that all OD cherry tomato samples remained acceptable, in terms of the main organoleptic attributes (Figure 5). Samples osmotically dehydrated in glycerol exhibited a pronounced sweetness that was accentuated by the addition of amino acids. Alanine-treated samples were associated with rich flavor, whereas glycine treatment resulted in a slight bitter aftertaste. Solutions containing oligofructose produced samples with a relatively neutral taste, while maltitol treatment led to a sour taste. The taste shifts observed for each treatment can be explained by the intrinsic taste properties of the incorporated solutes, taking also into account their interaction with the native sugar–acid balance of the tomato matrix. Glycerol itself possesses an inherent mild sweetness (relative sweetness ≈60–75% of sucrose) [57], which, combined with its low molecular weight and consequently higher molal uptake for a given mass gain (see Section 3.1), likely explains the pronounced sweet taste observed in glycerol-treated samples. This sweetness was further enhanced by the amino acids: glycine and alanine are themselves classified among the “sweet-tasting” amino acids at moderate concentrations [58,59], so their combination with glycerol is expected to produce an additive or synergistic sweetening effect, consistent with our sensory results. The slight bitter aftertaste noted for the glycine treatment can be attributed to the well-documented concentration dependence of amino acid taste quality: several free amino acids, including glycine, shift from a purely sweet profile at low concentration toward a mixed sweet/bitter or umami-like perception as concentration increases, due to concurrent activation of sweet and bitter taste receptor pathways [60,61]. Given the relatively high concentration of glycine applied here (18.6% *w*/*w*), such a concentration-dependent shift in taste quality is plausible. In contrast, oligofructose produced a comparatively neutral taste, consistent with its low relative sweetness (approximately 30–50% of sucrose) and mild flavor profile, properties that underlie its common use as a low-sweetness bulking agent in reduced-sugar food formulations [13]. Finally, the sour taste perceived in maltitol-treated samples is not attributable to an intrinsic sour taste of maltitol itself, which is typically described as sweet with only a mild cooling aftertaste [60]; rather, it more likely reflects a reduced masking of the tomato’s native organic acids (citric and malic acid) due to taste-mixture suppression effects, whereby the perceived intensity of sourness in a mixture depends on the relative intensity and temporal profile of the accompanying sweetness [61]. If maltitol’s sweetening power under the conditions applied here was insufficient to fully suppress the fruit’s intrinsic acidity—compared to the stronger, faster-onset sweetness contributed by glycerol—the tomato’s native sourness would be perceived as more dominant, explaining the sour notes reported by the sensory panel.

**Figure 6:**
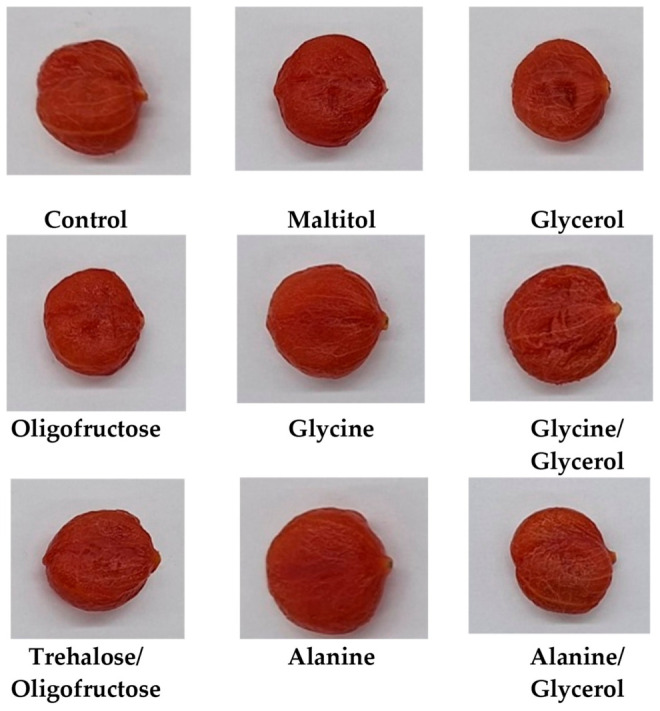
Pictures of non-treated and osmotically dehydrated (OD) cherry tomato samples at 35 °C for 120 min.

All osmotically treated samples exhibited increased firmness, compared to untreated tomatoes, with the highest firmness measured in alanine–glycerol-treated samples. Despite the increase in the overall color difference parameter (ΔE), perceptible visual color changes were minimal. Noticeable shrinkage and deformation were observed in samples treated with glycerol and glycine–glycerol solutions. Representative images of untreated and osmotically treated tomatoes are provided in Figure 6. Dermesonlouoglou et al. (2024) reported that OD (using glycerol as the main osmotic agent) frozen cherry tomatoes presented bright color, good texture and pleasant taste, whereas the untreated tomatoes suffered from a detrimental texture change (high drip loss and tissue softening) and taste deterioration during storage (from −5 °C to −23 °C) [11]. In the present work, the pre-treated samples were preferred in terms of all sensory attributes, including taste, and they showed increased stability during subsequent storage compared to non-treated samples Dermesonlouoglou et al. (2018) reported that panel assessments confirmed the analytical results of better-quality retention of osmodehydrofrozen kiwi fruits, also judging their flavor as pleasant (from −5 °C to −25 °C) [62]. Other research results showed that osmotic dehydration enhanced the sensory attributes of frozen fruits [32,63].

Future research should focus on the comprehensive characterization of the volatile profile of osmotically dehydrated tomatoes using instrumental analytical techniques, such as headspace solid-phase microextraction coupled with gas chromatography–mass spectrometry (HS-SPME/GC–MS) and/or electronic nose (E-nose) analysis. These approaches would provide objective information on aroma-active compounds and enable the correlation of instrumental measurements with sensory perception. Such investigations would contribute to a deeper understanding of the effects of different osmotic agents on flavor development and consumer acceptance, thereby complementing the findings of the present study.

### 3.3. Kinetics of OD Process

**Figure 7:**
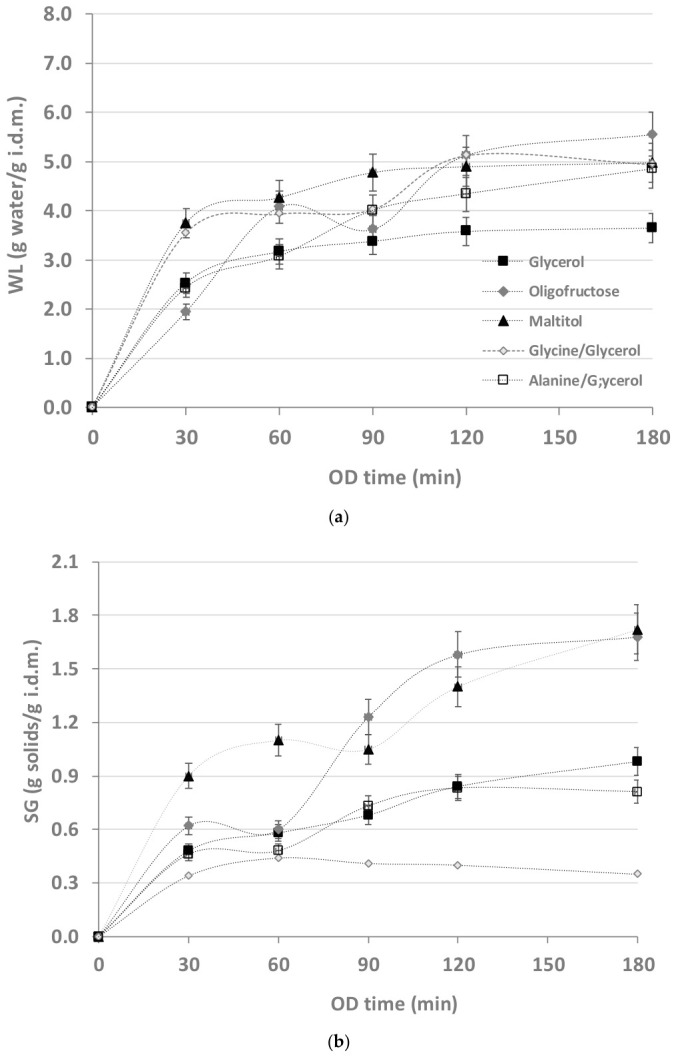
Evolution of (**a**) water loss (WL), and (**b**) solid gain (SG) of OD cherry tomatoes at 35 ° up to 180 min using (*w*/*w*) 50% glycerol, 50% oligofructose, 50% maltitol, 17% trehalose/33% oligofructose, 13.6% alanine, 18.6% glycine, 7% alanine/43% glycerol, 11% glycine/39% glycerol solution (Experimental data points: Average ± standard error).

The OD of cherry tomato samples at 35 °C was monitored from 30 min up to 180 min, in terms of water loss (WL) and solid gain (SG) (Figure 7). According to the Figures, the dehydration (water loss), as well as solids uptake, was accelerated with temperature increase. Water loss occurs rapidly during the initial stage (0–60 min), driven by the strong osmotic gradient between the sample and the solution [9]. Subsequently, the rate of water loss decreases and approaches a plateau that differs among the solutions, with final values ranging from 3.5 to 5.5 g H_2_O/g dry weight (d.w.). Although glycerol solutions were the most effective in reducing water activity, they resulted in comparatively lower water removal from the food matrix. With respect to solids uptake, a similar trend is observed. Mass transfer is most pronounced within the first 30 min, after which the rate gradually declines. Solutions containing glycerol led to lower solids uptake compared to those containing oligofructose and maltitol. Notably, tomatoes treated with the glycine–glycerol solution exhibited substantially lower solids uptake than those treated with the other solutions. The final solids uptake values ranged from 0.4 to 1.7 g solids/g d.w.

The progressive decline in the rate of WL and SG with increasing OD time reflects the diminishing driving force for mass transfer as the process approaches a pseudo-equilibrium state. As already discussed, mass transfer during OD is driven by the chemical potential (concentration) gradient between the intracellular fluid and the surrounding hypertonic solution; as water leaves the tissue and solutes penetrate it, this gradient progressively narrows, since the tissue’s internal solute concentration increases while, locally, the osmotic solution adjacent to the sample surface becomes diluted by the outgoing water [48,64]. Because a true thermodynamic equilibrium is reached only after very long immersion times, most practical OD processes—including the present one—are better described as approaching a pseudo-equilibrium condition within the studied time frame, a behavior widely captured by empirical kinetic models such as that of Azuara et al. [65], which explicitly model WL and SG as approaching asymptotic equilibrium values rather than increasing linearly with time.

In addition to the diminishing concentration gradient, structural changes in the plant tissue itself likely contribute to the observed plateau. As water loss and cell shrinkage proceed, the tissue undergoes progressive compaction and loss of turgor, reducing the effective porosity and cross-sectional area available for further mass transfer [65]. Furthermore, solute accumulation near the sample surface can promote a degree of “case hardening,” whereby a locally concentrated, more viscous solute layer forms at the tissue–solution interface, increasing the resistance to further water outflow and solute inflow and thereby slowing the process, particularly for the higher-molecular-weight, more viscous solutions (oligofructose, maltitol) tested in the present study [64]. This is consistent with the comparatively earlier plateau (after ~90 min) and lower final SG observed for the glycine–glycerol solution (Figure 7b, Table 2), where the combination of lower solute concentration and lower solution viscosity is expected to result in earlier attainment of pseudo-equilibrium and reduced case-hardening effects, relative to the higher-concentration sugar-based solutions.

Mass transport during osmotic dehydration was modeled using Equation (3). Modeling was carried out by fitting linear trend lines to WL–√t and SG–√t plots, where the slopes represent the respective rates of water loss and solids uptake. The calculated slopes are reported in Table 4, along with the coefficients of determination (R^2^), which indicate the goodness of fit of the linear models to the experimental data. Results indicate that water loss proceeded at a faster rate than solids uptake, with rates up to fourfold higher than those for solids incorporation. Analysis of the coefficients of determination (R^2^) further showed that solids uptake was more accurately described by the linear model than water loss. For the glycine–glycerol solution, the solids uptake rate stabilized after 90 min; therefore, only measurements obtained up to this time were considered in the rate estimation.

**Figure 8:**
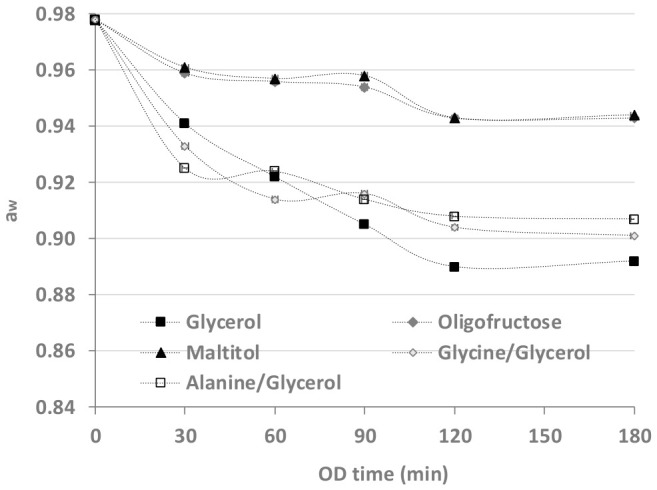
Evolution of water activity of cherry tomatoes during OD at 35 °C up to 180 min using (*w*/*w*) 50% glycerol, 50% oligofructose, 50% maltitol, 17% trehalose/33% oligofructose, 13.6% alanine, 18.6% glycine, 7% alanine/43% glycerol, 11% glycine/39% glycerol solution. Markers indicate experimental data points (average ± standard deviation).

As expected, water activity immediately decreases during the first minutes of the process and throughout its duration. According to Figure 8, the temperature significantly accelerates water activity decrease (*p* < 0.05), in agreement with published work [37]. A significant a_w_ reduction seems to be achieved at 45 °C and 60% glycerol solution (t = 180 min => a_w_ = 0.85). Water activity decreased exponentially over time, approaching a plateau between 120 and 180 min. Two distinct groups of osmotic solutions were identified: a more effective group, comprising glycerol-based treatments, a_w_ to approximately 0.90, and a less effective group, including maltitol and oligofructose solutions, which yielded higher final values around 0.94.

**Figure 9:**
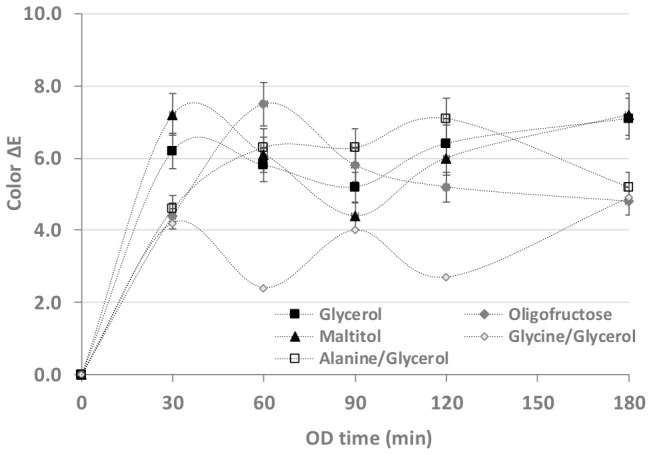
Evolution of color cherry tomatoes, ΔΕ, during OD at 35 °C up to 180 min using (*w*/*w*) 50% glycerol, 50% oligofructose, 50% maltitol, 17% trehalose/33% oligofructose, 13.6% alanine, 18.6% glycine, 7% alanine/43% glycerol, 11% glycine/39% glycerol solution. Markers indicate experimental data points (average ± standard deviation).

The color of the tomatoes exhibited noticeable changes from the initial minutes of the osmotic treatment, as indicated by ΔE > 2. Thereafter, no statistically significant differences were observed over the course of the process (*p* > 0.05). In contrast, comparisons among the different osmotic solutions revealed statistically significant differences (*p* < 0.05) with respect to color change. Post hoc analysis using Duncan’s multiple range test indicated that the glycine–glycerol solution differed significantly from the other treatments. The a/b ratio of the untreated samples was high, reflecting their intense red coloration, and remained at similarly elevated levels following osmotic dehydration. Furthermore, no statistically significant differences were observed among the different solutions (*p* > 0.05) (Figure 9).

**Figure 10:**
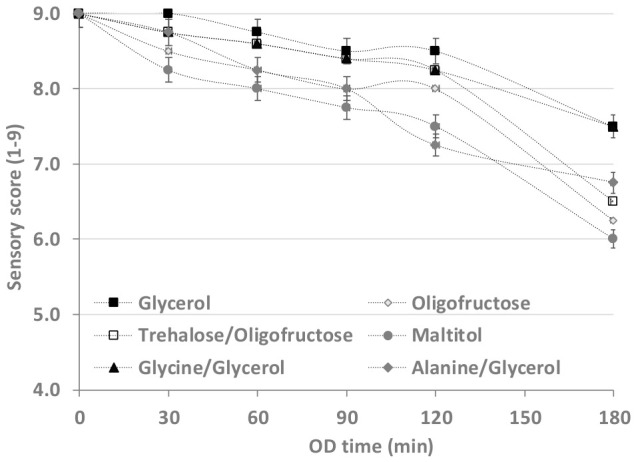
Sensory scores for cherry tomatoes during OD at 35 °C up to 180 min using (*w*/*w*) 50% glycerol, 50% oligofructose, 50% maltitol, 17% trehalose/33% oligofructose, 13.6% alanine, 18.6% glycine, 7% alanine/43% glycerol, 11% glycine/39% glycerol solution. Markers indicate experimental data points (average ± standard deviation).

Finally, the scores for the overall sensory quality/liking of OD cherry tomato samples at 35 °C from 30 min up to 180 min were presented (Figure 10).

### 3.4. Shelf-Life Estimation of OD-Treated Samples During Ultrachilled Storage

**Figure 11:**
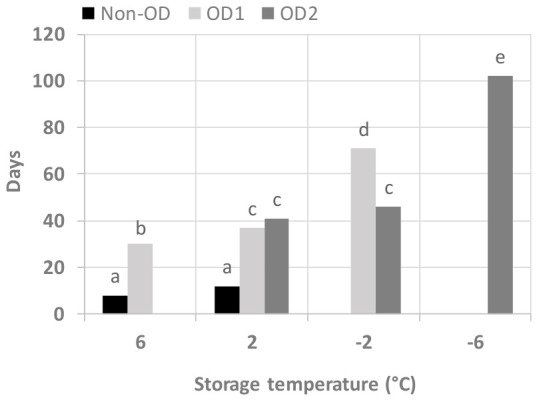
Shelf life (days) of non-OD and osmotically dehydrated (OD1 and OD2) cherry tomato samples at −6, −2, 2 and 6 °C, estimated by sensory testing (overall acceptability). Different letters (a–e) indicate significant differences, calculated by Duncan’s multiple range test for a significance level of *p* = 0.05.

In Figure 11, the shelf life of two different OD-treated cherry tomato samples was demonstrated (as a preliminary case study). Based on sensory evaluation results, osmotically treated samples (OD1 and OD2) exhibited significantly longer shelf life than the untreated ones, (non-OD) under the tested storage conditions. The shelf-life extension became more pronounced as storage temperature decreased. While the untreated samples exhibited a shelf life of only 8–12 days at refrigerated temperatures (6–2 °C), osmotically dehydrated samples remained stable for up to 30–41 days. Under ultrachilled conditions, shelf life increased further, reaching approximately 71 days for OD1 at −2 °C and 102 days for OD2 at −6 °C. Among OD treatments, OD2 exhibited the longest shelf life at all storage temperatures investigated, indicating a greater preservation effect than OD1, attributed to a more effective reduction in water activity and freezing point. The results demonstrate the synergistic effect of osmotic dehydration and ultrachilled storage on product stability and preservation.

The extended shelf life observed under the above conditions suggests that this storage approach may offer an effective compromise between quality preservation and energy efficiency, particularly when combined with osmotic dehydration treatments that lower water activity and depress the freezing point of the product. The estimated cooling demand at −2 °C is approximately 36 kJ kg^−1^, increasing to 95 kJ kg^−1^ at −6 °C, and reaching 174 kJ kg^−1^ under conventional frozen storage conditions (−18 °C), where the latent heat of phase change is involved [66]. Accordingly, the energy requirement at −18 °C is nearly five times higher than that estimated for storage at −2 °C. Such reductions in cooling energy demand are important from both economic and environmental perspectives, given the increasing importance of energy-efficient food preservation technologies. Furthermore, storage in the ultrachilled range allows the product temperature to remain above its initial freezing point, leading to no ice formation within the tissue, thereby reducing cellular damage and contributing to improved retention of texture and overall product quality during storage [22,67].

The shelf-life extension observed for the OD-treated samples can be interpreted in terms of the well-established relationship between water activity and microbial growth. Most spoilage and pathogenic bacteria require a_w_ values above approximately 0.91 to grow, with a few notable exceptions such as *Staphylococcus aureus* (minimum a_w_ ≈ 0.86) and *Clostridium botulinum* (minimum a_w_ ≈ 0.93); most yeasts require a_w_ above ~0.88, while molds are the most tolerant, with some species capable of growth down to a_w_ ≈ 0.65–0.70 [68,69]. The a_w_ values achieved in the present study (0.8957–0.9173 for OD1 and OD2) therefore fall within a range that substantially restricts, but does not fully eliminate, the growth of common bacterial spoilage flora and several yeast species, while offering little inherent protection against xerotolerant molds. Consequently, the marked shelf-life extension observed—from 8–12 days for untreated samples to 30–41 days for OD-treated samples under refrigeration, and up to 71–102 days under ultrachilled conditions (Figure 11)—is best explained not by a_w_ reduction alone, but by the synergistic combination of reduced a_w_ with sub-refrigeration/ultrachilled storage temperatures, consistent with the “hurdle technology” concept, whereby multiple sub-lethal preservation factors act in combination to achieve a level of microbial control that none would achieve individually [68,70]. In this framework, the depressed IFP achieved through OD is what allows storage temperature itself to be lowered as an additional hurdle (down to −2 to −6 °C) without inducing ice formation, effectively adding a second preservation factor (low temperature) on top of the a_w_ reduction, without the quality loss associated with conventional freezing.

It should be noted that the shelf-life estimations reported here (Section 2.3) were based on sensory evaluation (overall acceptability) as sensory rejection is the limiting factor in osmotic minimally processed fruit products of this type. Microbial validation (total viable counts, yeast/mold counts) confirms microbiological spoilage acceptability during chilled storage of osmotically treated products [36,69] and could be even more so assumed for the ultrachilled storage conditions applied in this study. In extensive, multi-temperature shelf-life studies to be conducted for ultrachilled products in continuing research, such measurements will be included.

## 4. Conclusions

This study demonstrated that osmotic dehydration using alternative humectants effectively modified the physicochemical properties of peeled cherry tomatoes by reducing water activity and depressing the initial freezing point, while preserving acceptable sensory quality. Among the osmotic agents evaluated, glycerol-based formulations showed the greatest potential for enhancing process efficiency and extending product stability, although the selection of the osmotic medium should also consider its impact on sensory attributes. The combination of osmotic dehydration and ultrachilled storage proved to be a promising preservation strategy, as it substantially extended shelf life while potentially reducing freezing damage and energy requirements compared with conventional freezing. These findings support the application of osmotic dehydration as a mild pretreatment for the development of high-quality fruit and vegetable products with improved storage stability.

## Figures and Tables

**Table 1:** Composition of the alternative osmotic solutions (in all solutions, 3.5% NaCl and 1.5% CaCl_2_ was also added).

OD Solute	$Concentration\;(\%\frac{w}{w})$
Glycerol	40, 50, 60
Maltitol	30, 40, 50
Oligofructose	40, 50, 60
Trehalose	30
Oligofructose/Trehalose	33/17
Alanine–Glycerol	7/43
Glycine–Glycerol	11/39

**Table 2:** Sensory evaluation. Quality parameters and scale (9-point intensity/hedonic).

Sensory Attribute	Scale
Appearance	
Red color	Intensity, Redness (1 = yellow, 5 = red, 9 = brown)
Glossiness	Intensity, 1 = not perceptible, 9 = extremely intense
Deformation/Shrinkage	Intensity, 1 = not perceptible, 9 = extremely intense
Texture	
Firmness	Intensity, 1 = not perceptible, 9 = extremely intense
Juiciness	Intensity, 1 = not perceptible, 9 = extremely intense
Flavor	
Sweetness	Intensity, 1 = not perceptible, 9 = extremely intense
Sourness	Intensity, 1 = not perceptible, 9 = extremely intense
Aftertaste	Intensity, 1 = not perceptible, 9 = extremely intense
Tomato aroma	Intensity, 1 = not perceptible, 9 = extremely intense
Overall impression	Hedonic/Liking, 1 = extremely dislike to 9 = extremely like

**Table 3:** Initial freezing temperature (IFP, T_f_, °C) of OD cherry tomatoes at 35 °C for 120 min using (*w*/*w*) different osmotic solutes (40–60% glycerol, 30–50% maltitol, 40–60% oligofructose, 30% trehalose, 33% oligofructose + 17% trehalose, 7% alanine/43% glycerol, 11% glycine/39% glycerol vs. non-OD-treated) (Experimental data points: Average ± standard error).

OD Solute	${IFP\;(T}_{f})$
Non-OD	−1.060 ± 0.085 ^a^
Glycerol (40%)	−7.179 ± 0.835 ^b^
Glycerol (50%)	−7.312 ± 0.250 ^b^
Glycerol (60%)	−7.831 ± 0.247 ^b^
Maltitol (30%)	−3.450 ± 0.254 ^c^
Maltitol (40%)	−3.840 ± 0.457 ^cd^
Maltitol (50%)	−3.913 ± 0.267 ^cd^
Oligofructose (40%)	−3.110 ± 0.587 ^c^
Oligofructose (50%)	−3.450 ± 0.441 ^c^
Oligofructose (60%)	−3.889 ± 0.440 ^cd^
Trehalose (30%)	−3.508 ± 0.984 ^c^
Oligofructose (33%)/Trehalose (17%)	−3.852 ± 1.067 ^cd^
Maltitol (50%)	−4.118 ± 0.767 ^cd^
Alanine (7%)-Glycerol (43%)	−3.903 ± 0.170 ^cd^
Glycine (11%)-Glycerol (39%)	−5.871 ± 3.726 ^e^

Mean value ± standard deviation. Different superscript letters (a–e) indicate significant differences, calculated by Duncan’s multiple range test for a significance level of *p* = 0.05.

**Table 4:** Effective moisture diffusion and solids diffusion coefficients for different OD solutions, as calculated from Equation (3), after being fitted to the experimental data.

OD Solute	$k_{WL}\left(\frac{g_{H_{2}O}}{g_{d.w.}\;\cdot \;s^{0.5}}\right)$	R^2^	$k_{SG}\left(\frac{g_{solids}}{g_{d.w.}\;\cdot \;s^{0.5}}\right)$	R^2^
Glycerol (50%)	0.332 ± 0.026 ^c^	0.830	0.074 ± 0.002 ^a^	0.982
Oligofructose (50%)	0.434 ± 0.023 ^ab^	0.894	0.125 ± 0.008 ^b^	0.922
Maltitol (50%)	0.460 ± 0.038 ^b^	0.801	0.129 ± 0.039 ^b^	0.955
Alanine (7%) –Glycerol (43%)	0.384 ± 0.033 ^a^	0.961	0.069 ± 0.001 ^a^	0.932
Glycine (11%)–Glycerol (39%)	0.4380 ± 0.015 ^ab^	0.850	0.059 ± 0.004 ^c^	0.997

±Standard error. Different superscript letters in the same column indicate significant differences, calculated by Duncan’s multiple range test for a significance level of *p* = 0.05.

## Data Availability

The original contributions presented in this study are included in the article. Further inquiries can be directed to the corresponding author.
